# The Chaperone-Dependent Ubiquitin Ligase CHIP Targets HIF-1α for Degradation in the Presence of Methylglyoxal

**DOI:** 10.1371/journal.pone.0015062

**Published:** 2010-11-29

**Authors:** Carla Figueira Bento, Rosa Fernandes, José Ramalho, Carla Marques, Fu Shang, Allen Taylor, Paulo Pereira

**Affiliations:** 1 Center of Ophthalmology and Vision Sciences (COCV), Institute for Biomedical Research in Light and Image (IBILI), Faculty of Medicine, University of Coimbra, Coimbra, Portugal; 2 Experimental Biology and Biomedicine (BEB) PhD Programme, Center for Neuroscience and Cell Biology (CNC), University of Coimbra, Coimbra, Portugal; 3 Jean Mayer USDA Human Nutrition Research Center on Aging, Tufts University, Boston, Massachusetts, United States of America; Chinese University of Hong Kong, Hong Kong

## Abstract

Hypoxia-inducible factor-1 (HIF-1) plays a key role in cell adaptation to low oxygen and stabilization of HIF-1 is vital to ensure cell survival under hypoxia. Diabetes has been associated with impairment of the cell response to hypoxia and downregulation of HIF-1 is most likely the event that transduces hyperglycemia into increased cell death in diabetes-associated hypoxia. In this study, we aimed at identifying the molecular mechanism implicated in destabilization of HIF-1 by high glucose. In this work, we identified a new molecular mechanism whereby methylglyoxal (MGO), which accumulates in high-glucose conditions, led to a rapid proteasome-dependent degradation of HIF-1α under hypoxia. Significantly, MGO-induced degradation of HIF-1α did not require the recruitment of the ubiquitin ligase pVHL nor did it require hydroxylation of the proline residues P402/P564 of HIF-1α. Moreover, we identified CHIP (Carboxy terminus of Hsp70-Interacting Protein) as the E3 ligase that ubiquitinated HIF-1α in the presence of MGO. Consistently, silencing of endogenous CHIP and overexpression of glyoxalase I both stabilized HIF-1α under hypoxia in the presence of MGO. Data shows that increased association of Hsp40/70 with HIF-1α led to recruitment of CHIP, which promoted polyubiquitination and degradation of HIF-1α. Moreover, MGO-induced destabilization of HIF-1α led to a dramatic decrease in HIF-1 transcriptional activity. Altogether, data is consistent with a new pathway for degradation of HIF-1α in response to intracellular accumulation of MGO. Moreover, we suggest that accumulation of MGO is likely to be the link between high glucose and the loss of cell response to hypoxia in diabetes.

## Introduction

Cell response to ischemia is primarily regulated by the transcription factor HIF-1 (hypoxia-inducible factor-1) [1] that triggers protective and adaptive mechanisms, promoting cell survival under hypoxia. Thus, any mechanism that destabilizes HIF-1 has a negative impact on cell adaptation to hypoxia. HIF-1 is a heterodimer composed of two subunits: a labile HIF-1α subunit and a stable HIF-1β subunit. Under normoxia, HIF-1α is hydroxylated on prolines 402 and 564 in the oxygen dependent degradation domain (ODD) by specific prolyl hydroxylases. Once hydroxylated, HIF-1α binds to the von Hippel Lindau protein (pVHL), which is part of an E3 ligase complex, resulting in HIF-1α polyubiquitination and subsequent proteasomal degradation [2], [3], [4]. In addition, asparagine 803 is also hydroxylated inhibiting the interaction of HIF-1α with the co-activator p300, leading to further repression of HIF-1 transcriptional activity [5]. When oxygen becomes limiting, the proline residues are not hydroxylated and HIF-1α escapes degradation, accumulating in the cell. HIF-1α is imported into the nucleus, dimerizes with HIF-1β and binds to hypoxia responsive elements (HREs), enabling transcriptional activation of more than 70 genes that help cells to cope and survive under hypoxia [1], [6], such as the vascular endothelial growth factor (VEGF).

Recently, it was shown that diabetes and hyperglycemia leads to downregulation of HIF-1 [7], [8], [9]. For example, downregulation of HIF-1 in response to hyperglycemia is likely to account for the decreased arteriogenic response triggered by myocardial ischemia in diabetic patients [10], [11]. Moreover, blood glucose levels were shown to vary in linear relation with fatal outcome after an acute hypoxic challenge, suggesting a deleterious influence of hyperglycemia on the ability of tissues to adapt to low oxygen [12]. In addition, levels of HIF-1 were found to be downregulated in biopsies from ulcers of diabetic patients as compared to venous ulcers that share the same hypoxic environment but are not exposed to hyperglycemia [7]. These and other evidences strongly suggest that cell and tissue dysfunction associated with diabetes is related, at least in part, with loss of cell response to hypoxia. However, the molecular mechanisms underlying this dysfunction remain to be elucidated.

Herein we hypothesize that increased production of methylglyoxal (MGO) is the link between high glucose and destabilization of HIF-1 in diabetes. Methylglyoxal (MGO) is a highly reactive α-oxoaldehyde formed as a by-product of glycolysis [13], [14]. Indeed, high glucose leads to intracellular accumulation of MGO in several tissues and increased concentration of MGO in cells and tissues has been implicated in the pathophysiology of a variety of diseases, including many diabetic complications [13], [14]. MGO is known to react with the free amino groups of lysine and arginine residues, leading to the formation of advanced glycation end products (AGEs) [13], and increased levels of MGO have deleterious effects in a number of essential signaling pathways [15], [16]. Of significance, AGEs were shown to impair the angiogenic process in a model of ischemia-induced retinopathy [17].

Data presented in this paper shows that MGO was able to induce the degradation of HIF-1α and to decrease the transcriptional activity of HIF-1. The MGO-induced destabilization of HIF-1α did not involve recruitment of the pVHL ubiquitin ligase nor did it require hydroxylation of the prolines residues P402/P564 of HIF-1α. We identified CHIP (Carboxyl terminus of the Hsc70-Interacting Protein) as the ubiquitin ligase that targets HIF-1α for degradation in the presence of MGO, by a mechanism that requires prior recruitment of the molecular chaperones Hsp40 and Hsp70.

## Results

### Intracellular accumulation of MGO decreased the half-life of HIF-1α

**Figure 1:**
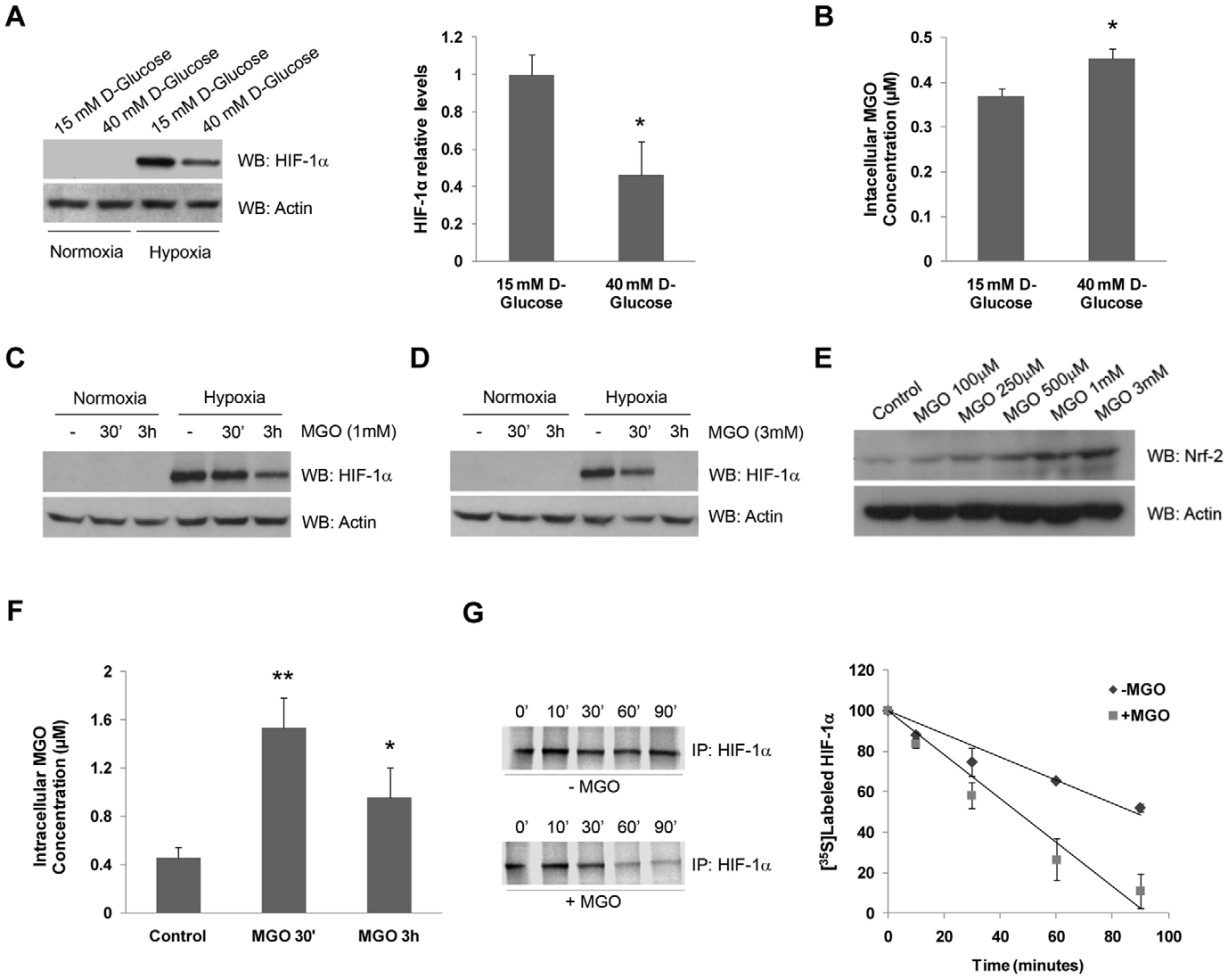
High glucose induced intracellular accumulation of methylglyoxal, which in turn led to decreased levels of HIF-1α. (A) ARPE-19 cells were grown in 15 mM (basal DMEM: F12 medium) or 40 mM of D-glucose during 10 days. During the last 61 hours of incubation, cells were exposed to hypoxia (2% O_2_). After the treatments, the proteins were separated by SDS-PAGE, transferred to PVDF membranes and probed against HIF-1α and actin. The results represent the mean ± SD of at least three independent experiments. * p<0.05, significantly different from control (t test). (B) ARPE-19 cells were grown in DMEM: F12 medium containing 15 mM or 40 mM of D-glucose during 10 days. Cells were then lysed in acetic acid (0.1 M) and the intracellular levels of MGO were determined by HPLC analysis after derivatization with DDB. The results represent the mean ± SD of at least three independent experiments. * p<0.05, significantly different from control (t test). (C and D) ARPE-19 cells were exposed to hypoxia (2% O_2_) for 6 hours and MGO (1 mM or 3 mM) was added for 30 minutes or 3 hours. The cell lysates were analyzed by immunoblot using antibodies for HIF-1α and actin. (E) ARPE-19 cells were treated with different MGO concentrations (100 µM to 3 mM) for 2 hours. The cell lysates were analyzed by western blot using antibodies for Nrf-2 and actin. (F) ARPE-19 cells were treated with 3 mM of MGO for 30 minutes or 3 hours. Cells were then lysed in acetic acid (0.1 M) and the intracellular levels of MGO were determined by HPLC after derivatization with DDB. The results represent the mean ± SD of at least three independent experiments. * p<0.05 and ** p<0.01, significantly different from control (one-way ANOVA with the Dunnet's comparison test). (G) After metabolic labeling with L-[^35^S] methionine/cysteine under hypoxia, ARPE-19 cells were maintained under hypoxia, either in the absence or the presence of MGO (3 mM). Cells were harvested at 0, 10, 30, 60 and 90 minutes in 0.5% NP-40 lysis buffer. HIF-1α was immunoprecipitated and radiolabeled HIF-1α protein was assessed by SDS-PAGE and autoradiography.

Hyperglycemia was shown to be involved in the loss of cell response to hypoxia in diabetes, through a mechanism that is likely to involve downregulation of HIF-1α. Indeed, data shows that levels of HIF-1α were downregulated under hypoxia in cells treated with high glucose over a period of 10 days, as compared to cells maintained in basal glucose concentration (Figure 1A). In this study, we used a retinal pigment epithelial cell line (ARPE-19) to investigate the role of high glucose in regulation of HIF-1. Retinal pigment epithelial (RPE) cells are a good model to evaluate cell response to hypoxia since these cells are extremely sensitive to variations in oxygen [18] and glucose concentrations [19] and are a major source of VEGF in the retina, a gene well known to be upregulated by hypoxia [6], [20].

To further emphasize the relevance of high glucose in downregulation of HIF-1α, we assessed the intracellular levels of MGO following exposure of ARPE-19 cells to high glucose. Data shows that after 10 days of incubation with high glucose (40 mM), the intracellular levels of MGO increased by about 23%, reaching the concentration of 0.45±0.024 µM (Figure 1B). Consistently, Figures 1C and 1D show that treatment of cells with MGO induced destabilization of HIF-1α, which was previously accumulated under hypoxia, in a dose and time-dependent manner. After 30 minutes of incubation with 3 mM MGO there was a decrease of about 50% in the levels of HIF-1α, whereas after 3 hours of incubation HIF-1α was virtually undetectable (Figure 1D). As a control, we show that the effect of MGO cannot be generalized to all transcription factors. Indeed, MGO did not downregulate other transcription factors, such as NF-E2 related factor 2 (Nrf-2) (Figure 1E). Moreover, incubation of RPE cells with 3 mM MGO for 30 minutes or for 3 hours resulted in an intracellular accumulation of MGO of 1.54±0.246 µM or 0.96±0.247 µM, respectively (Figure 1F). The decrease of MGO after 3 hours of incubation probably reflects scavenging by glyoxalases [21], [22], as well as irreversible binding of MGO to proteins and other cell components [14]. The range of MGO concentrations used in this work have been widely used in other studies [15], [23] and result in intracellular concentrations of MGO that are within the physiological range reported in pathological conditions, such as diabetes [24], [25], [26], [27].

Additionally, pulse-chase experiments clearly showed that MGO decreased the half-life of HIF-1α by about two fold, from 98 minutes to 47 minutes (Figure 1G). This indicates that MGO did indeed downregulate HIF-1α by promoting an increased degradation of the protein.

**Figure 2:**
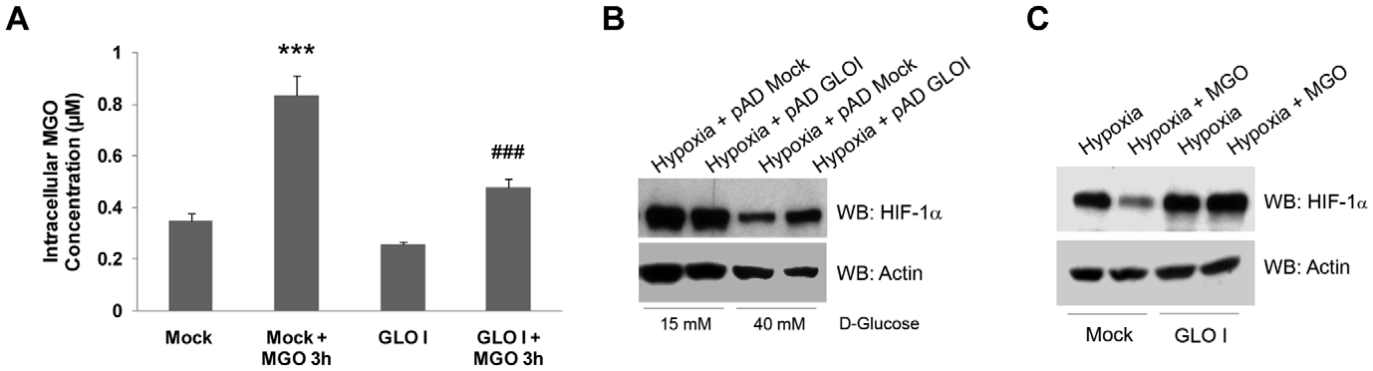
Glyoxalase I overexpression significantly stabilized HIF-1α under high glucose or methylglyoxal treatment. (A) ARPE-19 cells were infected with pAD hGLOI adenovirus for 48 hours. By the end of infection, cells were treated with 3 mM of MGO for 3 hours and were, subsequently, lysed in acetic acid (0.1 M). The intracellular levels of MGO were determined by HPLC after derivatization with DDB. The results represent the mean ± SD of at least three independent experiments. *** p<0.001, significantly different from Mock; ### p<0.001, significantly different from Mock + MGO3h (one-way ANOVA with Tukey's multiple comparison test). (B) ARPE-19 cells were grown in 15 mM (basal DMEM: F12 medium) or 40 mM of D-glucose during 10 days. After 8 days of incubation, cells were infected with pAd hGLOI adenovirus for 48 hours. During the last 6 hours of incubation, cells were subjected to hypoxia and subsequently lysed. Proteins were separated by SDS-PAGE, transferred to PVDF membranes and analyzed by immunoblot against HIF-1α and actin. (C) ARPE-19 cells were infected with pAD hGLOI adenovirus for 48 hours. During the last 6 hours of incubation, cells were subjected to hypoxia in the presence of MGO (3 mM for 3 hours). Subsequently, cells were lysed and the proteins were separated by SDS-PAGE, transferred to PVDF membranes and probed against HIF-1α and actin. The word “Mock” in the figures refers to a control with an empty vector.

Significantly, overexpression of glyoxalase I (GLO I), the rate-limiting enzyme involved in the detoxification of MGO, prevented intracellular accumulation of MGO (Figure 2A) and promoted stabilization of HIF-1α under hypoxia following treatment with either high glucose (Figure 2B) or MGO (Figure 2C). Although we cannot exclude that other putative AGE precursors are also involved in HIF-1α destabilization under high glucose, these data clearly support a role for MGO in destabilization of HIF-1α under high glucose. Indeed, overexpression of GLO I stabilized HIF-1α (Figure 2B), which indicates that an increase of about 23% in the intracellular levels of MGO (Figure 1B) appears to be sufficient to promote a strong decrease on the levels of HIF-1α.

### MGO induced a decrease of the HIF-1 transcriptional activity

**Figure 3:**
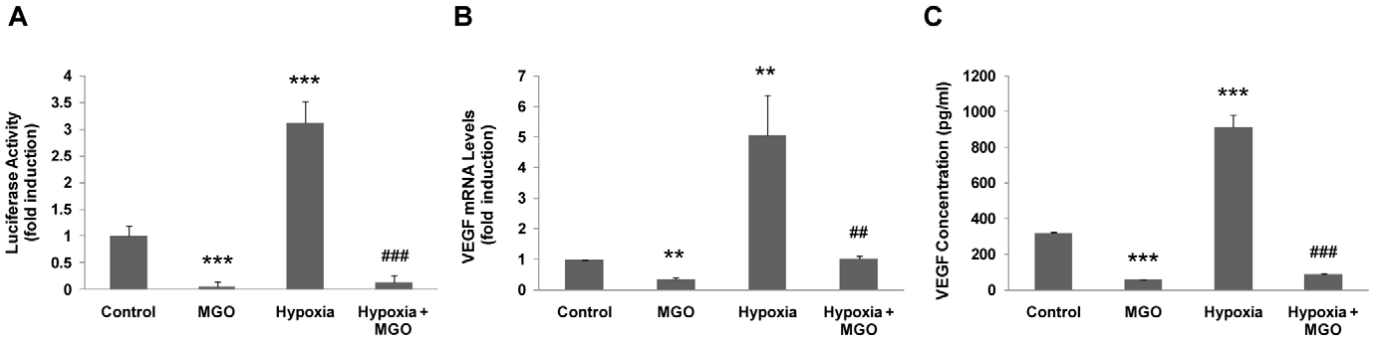
Methylglyoxal decreased the transcriptional activity of HIF-1 and the release of VEGF_121/165_ into the medium. (A) ARPE-19 cells were transiently transfected with the pT81 HRE-luciferase vector and were subjected to hypoxia (2% O_2_) for 6 hours in the absence or presence of MGO (1 mM for 4 hours). Subsequently, the luciferase activity was determined and the values were expressed as fold induction over control. (B) ARPE-19 cells were subjected to hypoxia (2% O_2_) for 6 hours either in the absence or the presence of MGO (1 mM for 4 hours). Total RNA was used to synthesize cDNA, which, in turn, was used as template to quantify VEGF mRNA and 18S rRNA through RT-PCR. (C) ARPE-19 cells were subjected to hypoxia (2% O_2_) for 6 hours either in the absence or in the presence of MGO (1 mM for 4 hours). The concentration of the diffusible VEGF_121_ and VEGF_165_ isoforms were determined by ELISA using a monoclonal antibody for human VEGF. The results represent the mean ± SD of at least three independent experiments. ** p<0.01 and *** p<0.001, significantly different from control; ## p<0.01 and ### p<0.001, significantly different from hypoxia condition (one-way ANOVA).

The downregulation of HIF-1α protein levels following exposure to MGO consistently led to a decrease in HIF-1 transcriptional activity. To initiate transcription, HIF-1 binds to the HREs of a number of target genes, including VEGF. Data represented in Figure 3A shows that hypoxia led to a significant increase in HIF-1 transcriptional activity as evaluated by an HRE-luciferase associated reporter gene assay. Incubation with MGO dramatically decreased the activity of luciferase, suggesting a strong decrease in HIF-1 transcriptional activity. Consistent with this decrease in the transcriptional activity, the levels of VEGF mRNA also decreased in the presence of MGO as compared to hypoxic cells cultured in the absence of MGO (Figure 3B). The levels of VEGF secreted into the culture medium also decreased as compared to cells subjected only to hypoxia (Figure 3C). We believe that the observed decrease on the transcriptional activity of HIF-1 is, most likely, due to changes on the total amount of the HIF-1α protein, rather than effective decreased activity of HIF-1 to transactivate its target genes and activate the transcriptional machinery. However, this effect may not be excluded.

The downregulation of HIF-1 transcriptional activity under normoxia following treatment with MGO can be ascribed to low, but detectable, HIF-1α levels in controls, which further decreased after treatment with MGO (as revealed by longer exposures of immunoblot membranes).

### MGO induced polyubiquitination and proteasome-dependent degradation of HIF-1α

**Figure 4:**
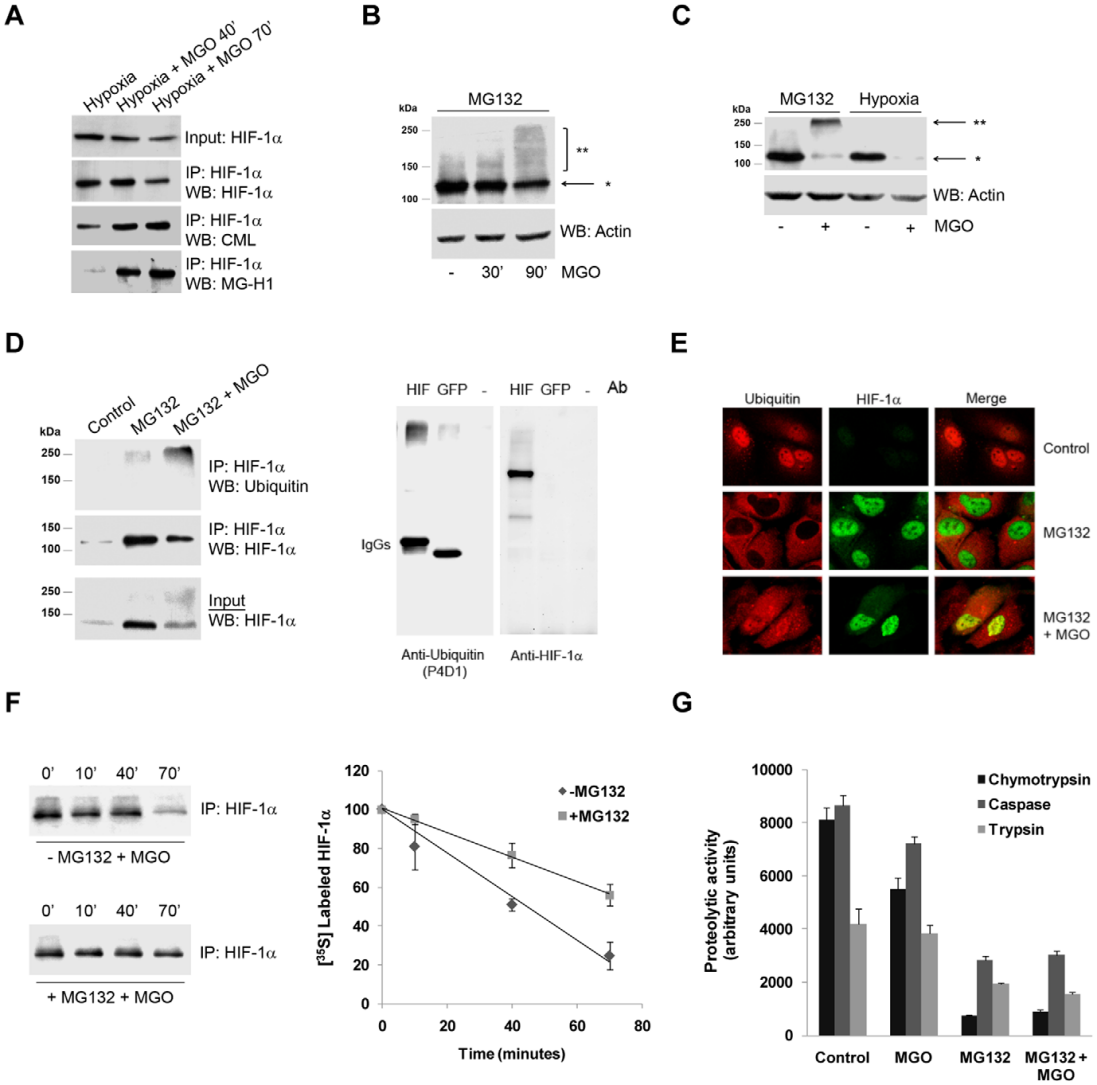
Methylglyoxal modified HIF-1α and induced its polyubiquitination. (A) ARPE-19 cells were subjected to hypoxia (2% O_2_) for 6 hours and then incubated with MGO (3 mM) for 40 and 70 minutes. HIF-1α was immunoprecipitated and the immunoprecipitates were probed against CML and MG-H1. (B and C) ARPE-19 cells were treated with MG132 (20 µM) for 4 hours or subjected to hypoxia (2% O_2_) for 6 hours and then incubated with MGO (3 mM) for 30 minutes, 90 minutes (B) or 3 hours (C). The cell lysates were analyzed by immunoblot against HIF-1α and the PVDF membranes were overexposed to reveal higher molecular weight bands; * HIF-1α of 120 kDa; ** posttranslationally modified HIF-1α (with higher molecular weights). (D) ARPE-19 cells were treated with MG132 (20 µM) for 4 hours either in the presence or absence of MGO (3 mM) for the last 90 minutes. HIF-1α was immunoprecipitated and immunoprecipitates were probed against HIF-1α and ubiquitin (P4D1). IP controls were carried out both with no antibody and with an irrelevant mouse IgG1 antibody (anti-GFP). (E) ARPE-19 cells cultured in coverslips were transfected with HA tagged HIF-1α. Subsequently, cells were treated with MG132 (20 µM) for 4 hours in the absence or presence of MGO (3 mM) for 90 minutes. Cells were fixed with 4% PFA for 10 minutes and used for immunocytochemistry using specific antibodies directed against HA and ubiquitin (FK1). (F) ARPE-19 cells were incubated with L-[^35^S] methionine/cysteine under hypoxia. After metabolic labeling, cells were maintained under hypoxia, in the presence of 3 mM of MGO and either in the absence or presence of MG132 (20 µM). Cells were harvested at 0, 10, 40 and 70 minutes in 0.5% NP-40 lysis buffer. HIF-1α was immunoprecipitated and radiolabeled HIF-1α protein was assessed by SDS-PAGE and autoradiography. (G) ARPE-19 cells were treated with the proteasome inhibitor MG132 (20 µM) for 4 hours, either in the absence or presence of MGO (3 mM for 3 hours). The 20S proteasome activities were determined by *in vitro* fluorogenic assays, using specific substrates for each activity: Suc-LLVY-MCA (chymotrypsin-like activity), Z-LLE-MCA (caspase-like activity) and Boc-LRR-MCA (trypsin-like activity). The values reported in the graph correspond to measurements at 30 minutes of activity.

MGO has recently been shown to modify HIF-1α on arginine residues [22], probably leading to changes in protein conformation. Indeed, immunoprecipitation experiments showed that MGO modified lysine and arginine residues of HIF-1α, increasing its immunoreactivity against Nε-carboxymethyl-lysine (CML) and Nα-acetyl-Nδ(5-hydro-5-methyl)-4-imidazolone (MG-H1) antibodies, respectively (Figure 4A). Thus, we hypothesized that modification by MGO might stimulate proteasome-dependent degradation of HIF-1α, as a result of post-translational modifications.

Treatment of cells with proteasome inhibitors, following incubation with MGO, resulted in the accumulation of high molecular weight bands that immunoreacted with anti-HIF-1α antibodies (Figure 4B). It is interesting to note that the pattern of HIF-1α ubiquitination in the presence of MGO varied in a time-dependent manner. Indeed, for the initial time points a characteristic high-molecular weight ladder can be observed (Figure 4B). However, this ladder disappeared for longer incubations, which was accompanied by the accumulation of higher molecular weight forms of HIF-1α on the top of the gel (Figure 4C). These observations were likely to result from a gradual and time-dependent ubiquitination of the protein in response to MGO treatment, resulting in the accumulation of increasingly higher molecular weight forms of the protein. It should also be noted that the effect of MGO under hypoxia is quite different from the effect observed under proteasome inhibition (compare lane 2 with lane 4 on Figure 4C). Indeed, under hypoxia and MGO, HIF-1α was readily degraded, whereas in the presence of MG132 there was an apparent loss of HIF-1α (120 kDa), with an accumulation of high molecular weight forms of the protein that corresponded to ubiquitinated forms of HIF-1α, as confirmed by immunoprecipitation experiments (Figure 4D). It should be noted that the lysis buffer used in the IPs contained a low percentage of a non-ionic detergent (0.5% NP-40) with low solubility potential in contrast to the 2x Laemmli buffer used to prepare whole cell lysates such as the data presented in Figures 4B and 4C. This may explain the poor detection of high molecular weight bands, in the presence of MGO, in the panel corresponding to the input samples of the IP experiments.

Previous results suggested that MGO-induced degradation of HIF-1α is proteasome-dependent and involves ubiquitination of the protein. Immunofluorescence data further confirmed these results. Data shows that proteasome inhibition *per se* decreased the levels of polyubiquitin conjugates in the nucleus, consistent with previous reports [28], [29]. However, addition of MGO changed the distribution of ubiquitin in the cell, strongly increasing the nuclear co-localization between ubiquitin conjugates and HIF-1α (Figure 4E). We further show that the proteasome inhibitor MG132 (or epoxomicin) prevented MGO-induced degradation of HIF-1α and extended the half-life of the protein by about 1.7 fold (Figure 4F). Taken together, these observations indicate that HIF-1α was modified by MGO and that such modification resulted in ubiquitin-dependent degradation of the protein. However, because proteasome inhibitors did not fully prevent HIF-1α degradation, it seems reasonable to suggest that other degradation pathways are likely to be involved in HIF-1α degradation induced by MGO. An alternative explanation is that, even in the presence of inhibitors such as MG132, a residual proteasomal activity was maintained (Figure 4G), which might be sufficient to sustain some level of degradation of HIF-1α in the presence of MGO.

### Independence of VHL and proline hydroxylation in the MGO-induced destabilization of HIF-1α

**Figure 5:**
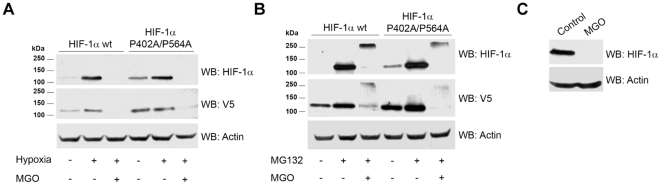
Destabilization of HIF-1α induced by methylglyoxal was independent on VHL. (A and B) ARPE-19 cells were transiently transfected with HIF-1α wt-V5 or HIF-1α (P402A/P564A)-V5 plasmids. Cells were subsequently subjected to hypoxia (2% O_2_) for 6 hours (A) or treated with MG132 (20 µM) for 4 hours (B), in the absence or presence of MGO (3 mM for 3 hours). The cell lysates were immunoblotted against HIF-1α and V5. (C) RCC4 VHL^-/-^ cells were treated with MGO (3 mM for 3 hours) and cell lysates were analyzed by western blot for HIF-1α and actin.

The canonical pathway for HIF-1α degradation requires recruitment of the ubiquitin ligase pVHL and prior hydroxylation of prolines 402 and 564 on HIF-1α. ARPE-19 cells were transfected with a mutant HIF-1α where the proline residues 402 and 564 were mutated to alanine (P402A/P564A). Results presented in Figure 5A show that mutant HIF-1α was readily degraded in the presence of MGO indicating that prolines 402 and 564 were not required for MGO-induced degradation of HIF-1α. Moreover, both WT and mutated HIF-1α accumulated as high molecular weight bands in the presence of proteasome inhibitors and MGO, indicating that the mechanism whereby mutant HIF-1α is degraded in the presence of MGO did not require proline hydroxylation but was still dependent on the proteasome (Figure 5B). To further confirm that this new pathway is independent on pVHL, a VHL deficient cell line (RCC4 VHL^−/−^) was treated with MGO. Results in Figure 5C show that in untreated cells, HIF-1α was inherently stable, whereas treatment with MGO resulted in the destabilization and rapid degradation of the transcription factor.

### CHIP induced ubiquitination of HIF-1α in the presence of MGO

**Figure 6:**
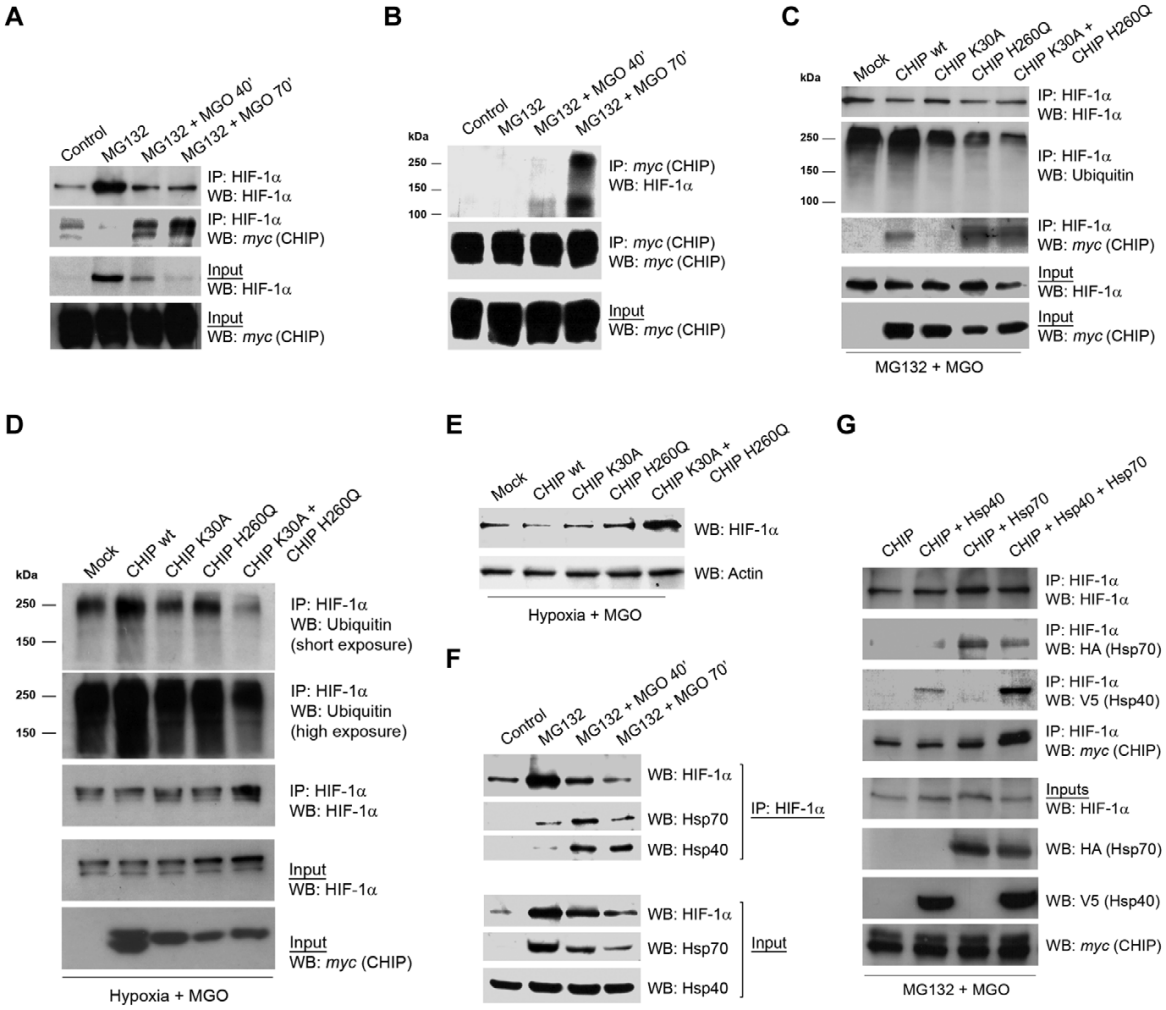
CHIP polyubiquitinated HIF-1α in the presence of methylglyoxal. (A and B) ARPE-19 cells were transiently transfected with the CHIP wt c-myc plasmid and treated with MG132 (20 µM) for 4 hours in the absence or presence of MGO (3 mM for 40 or 70 minutes). HIF-1α (A) or c-myc (B) were immunoprecipitated and the immunoprecipitates were probed for c-myc and HIF-1α. (C) ARPE-19 cells were transfected with CHIP wt c-myc or with the dominant negatives CHIP K30A c-myc and CHIP H260Q c-myc, simultaneously or separately, and treated with MG132 (20 µM) for 41 hours in the presence or absence of MGO (3 mM for 70 minutes). HIF-1α was then immunoprecipitated and the immunoprecipitates were blotted against HIF-1α, c-myc and ubiquitin (P4D1). (D) ARPE-19 cells were transfected with CHIP wt c-myc or with the dominant negative mutants CHIP K30A c-myc and CHIP H260Q c-myc, simultaneously or separately, and subjected to hypoxia for 6 hours in the presence or absence of MGO (3 mM for 70 minutes). HIF-1α was then immunoprecipitated and the immunoprecipitates were blotted for HIF-1α and ubiquitin (P4D1). (E) ARPE-19 cells were transfected with CHIP wt c-myc, CHIP K30A c-myc or/and CHIP H260Q c-myc dominant negatives, either simultaneously or separately, and subjected to hypoxia for 6 hours in the presence or absence of MGO (3 mM for 70 minutes). Proteins were separated by SDS-PAGE, transferred to PVDF membranes and probed for HIF-1α and actin. (F) ARPE-19 cells were treated with MG132 (20 µM for 4 hours) either in the presence or absence of MGO (3 mM for 40 or 70 minutes). HIF-1α was immunoprecipitated and the immunoprecipitates were probed using antibodies against HIF-1α, Hsp70 and Hsp40. (G) ARPE-19 cells were transfected simultaneously with CHIP-c-myc and V5-tagged Hsp40 and/or HA-tagged Hsp70. Cells were subsequently treated with MG132 (20 µM) for 4 hours and MGO (3 mM) for the last 70 minutes of incubation. HIF-1α was immunoprecipitated and the immunoprecipitates were blotted against HA, V5 and c-myc. IP controls were carried out both with no antibody and with an irrelevant mouse IgG1 antibody (for example anti-GFP). The word “Mock” in the figures refers to a control with an empty vector.

MGO was likely to destabilize HIF-1α by inducing post-translational modification of the transcription factor, thus we suggested that ligases that target post-translational modified proteins were likely candidates to promote HIF-1α ubiquitination. Because CHIP is a chaperone-binding ligase that bridges the ubiquitin-proteasome pathway and molecular chaperones [30], [31], [32], we investigated whether CHIP was the ligase responsible for HIF-1α ubiquitination in the presence of MGO. ARPE-19 cells were transfected with c-myc CHIP and subsequently treated with the proteasome inhibitor MG132 (or epoxomicin) in the presence or the absence of MGO. Data in Figure 6A shows that treatment with MGO increased the association between CHIP and HIF-1α. In the absence of MGO there was also evidence for a limited interaction between both proteins, suggesting a potential role for CHIP in degradation of HIF-1α under normoxia. However, under physiological conditions and normoxia, the large majority of HIF-1α in cells is presumably degraded via VHL-dependent proteolysis and the physiological function of CHIP-mediated degradation of HIF-1α is, at the moment, unclear.

Following treatment with MGO and proteasome inhibitors, HIF-1α that co-immunoprecipitated with CHIP migrated at higher molecular weights (Figure 6B), consistent with ubiquitinated forms of the protein. Significantly, overexpression of CHIP in the presence of MGO revealed an accumulation of polyubiquitinated forms of HIF-1α (Figures 6C and 6D). Moreover, CHIP mutants that either do not bind chaperones (K30A) or do not present ubiquitin ligase activity (H260Q) decreased the ubiquitination of HIF-1α (Figures 6C and 6D) and partially stabilized the protein (Figure 6E), above the control levels. Moreover, data consistently shows that CHIP K30A did not co-immunoprecipitate with HIF-1α, while both wild type CHIP and the mutant CHIP H260Q bound to and co-precipitated with HIF-1α (Figure 6C). Simultaneous overexpression of the two mutants was more effective in stabilizing HIF-1α, than individual overexpression of each mutant (Figure 6E). Consistent with our model, this is likely to reflect the need for both ubiquitin ligase activity and binding of chaperones to trigger CHIP-mediated degradation of HIF-1α in the presence of MGO. One may speculate that the synergistic effect of the two mutants was related to their specific mechanisms of action and competition with endogenous CHIP. For example, in the presence of both mutants, CHIP H260Q may block access of endogenous CHIP to its binding site on HIF-1α, thus contributing to stabilize the transcription factor. On the other hand, CHIP K30A may, in addition, sequester the E2 or other cofactors required for the activity of endogenous CHIP. In addition, overexpression of mutant CHIP is likely to favor the formation of non-functional CHIP dimers with endogenous CHIP [33]. This might explain the greater stabilization of HIF-1α in the presence of both mutants.

The observation that the mutant K30A CHIP was unable to bind and ubiquitinate HIF-1α suggests that binding to chaperones is essential for CHIP-dependent targeting and ubiquitination of HIF-1α. Indeed, results in Figure 6F show that treatment with MGO increased the association between Hsp40/70 and HIF-1α. We further observed that simultaneous overexpression of Hsp40 and Hsp70 increased the levels of CHIP associated with HIF-1α in the presence of MGO, suggesting that CHIP/HIF-1α association was mediated by Hsp40/Hsp70 (Figure 6G).

It should be noted that MGO-induced degradation of HIF-1α under hypoxia appeared to be very fast (Figure 1G), which made interaction between HIF-1α and the ancillary proteins difficult to detect. Considering that our data clearly shows that MGO-induced degradation of HIF-1α was independent on VHL and that the proteasome was still involved in CHIP-dependent degradation of HIF-1α in the presence of MGO, we performed a number of experiments using proteasome inhibitors, since this increased the half-life of HIF-1α (Figure 4F), allowing the detection of interactions between HIF-1α and binding partners, such as CHIP, Hsp40 and Hsp70 (Figures 6A, 6B, 6F, 6G).

**Figure 7:**
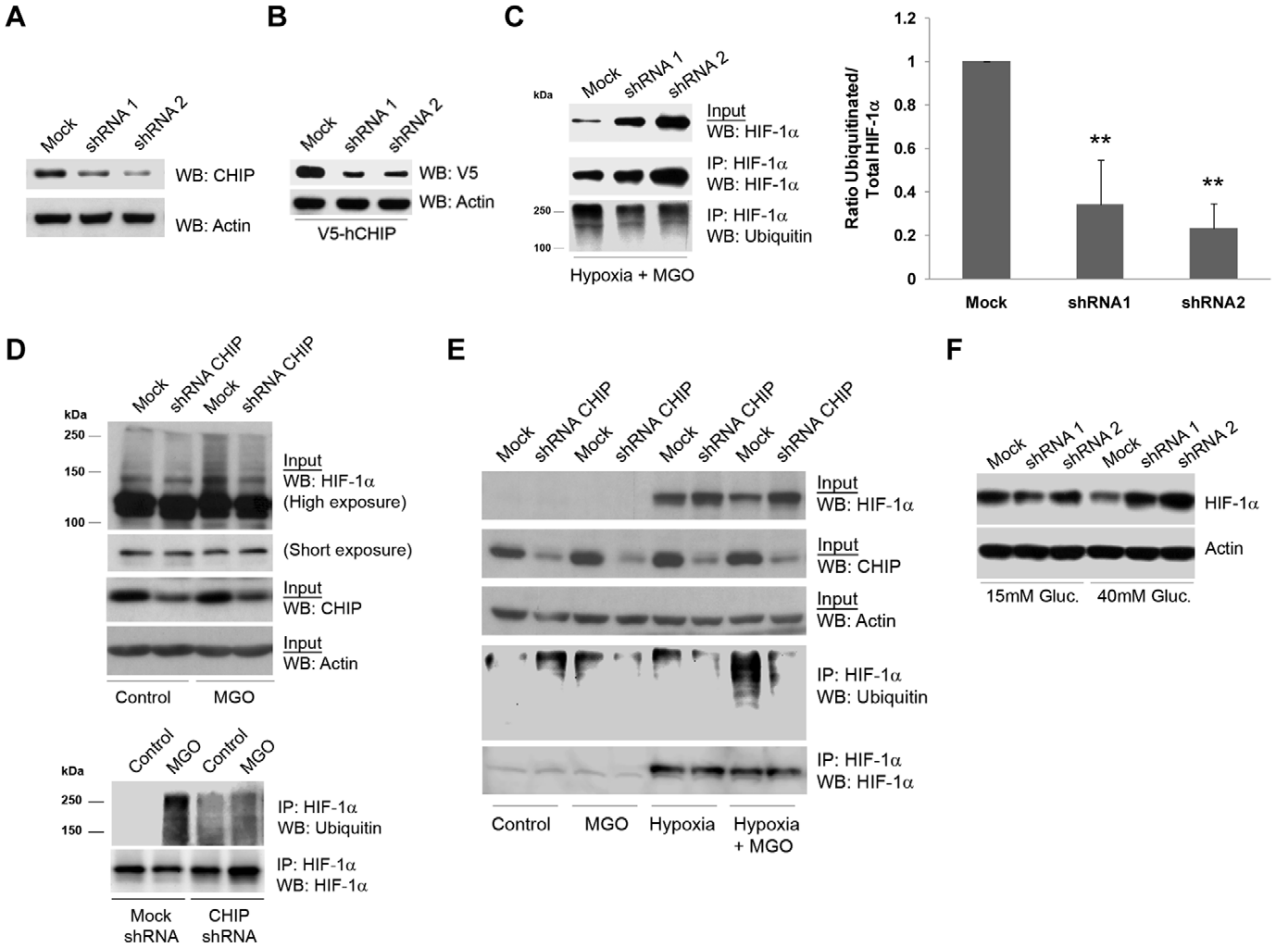
Silencing of CHIP stabilized HIF-1α protein in the presence of methylglyoxal in ARPE-19, Cos-7 and RCC4 cells. (A) ARPE-19 cells were infected with pAd shRNA-hCHIP for 48 hours. Cells were subsequently lysed and protein extracts were immunoblotted against endogenous CHIP and actin. (B) ARPE-19 cells were co-infected with pAd V5-hCHIP and pAd shRNA-hCHIP for 24 hours. The cell lysates were analyzed by western blotting using anti-V5 and anti-actin monoclonal antibodies. (C) ARPE-19 cells were infected with pAd shRNA-hCHIP for 48 hours and during the last 6 hours of incubation, cells were subjected to hypoxia in the presence of MGO (3 mM for the last 70 minutes). Cell lysates were used to immunoprecipitate HIF-1α and the immunoprecipitates were probed with antibodies against HIF-1α and ubiquitin (P4D1). The data in the graph represents the mean ± SD of at least three independent experiments. ** p<0.01, significantly different from control (one-way ANOVA with the Dunnet's comparison test). (D) RCC4 VHL^-/-^ cells, infected with pAd shRNA-hCHIP for 48 hours, were treated with MGO (3 mM for 70 minutes). Cell lysates were used to immunoprecipitate HIF-1α and the immunoprecipitates were blotted against HIF-1α and ubiquitin (P4D1). (E) Cos-7 cells were infected with pAd shRNA-hCHIP for 48 hours and, during the last 6 hours of incubation, cells were subjected to hypoxia and treated with MGO (3 mM for the last 70 minutes). Cell lysates were used to immunoprecipitate HIF-1α and the immunoprecipitates were probed for HIF-1α and ubiquitin (P4D1). IP controls were carried out both with no antibody and with an irrelevant mouse IgG1 antibody (for example anti-GFP). (F) ARPE-19 cells were grown in 15 mM (basal DMEM: F12 medium) or 40 mM of D-glucose during 10 days. After 8 days of incubation, cells were infected with pAd shRNA-hCHIP for 48 hours. During the last 6 hours of incubation, cells were subjected to hypoxia and subsequently lysed. Proteins were blotted against HIF-1α and actin. The word “Mock” in the figures refers to a control with a scrambled shRNA sequence.

Loss of function studies performed in various cell types, including ARPE-19, RCC4 VHL^−/−^ and Cos-7 cells, by silencing CHIP with shRNAs (Figure 7A and 7B), clearly showed that MGO-dependent degradation of HIF-1α is a general mechanism not restricted to one cell type and that is likely to have important biological functions. Indeed, depletion of CHIP by shRNAs decreased the accumulation of high molecular weight bands that cross-react with anti-HIF-1α antibodies (Figure 7D), decreased ubiquitination of HIF-1α and led to the stabilization of the protein under hypoxia in the presence of MGO in ARPE-19 (Figures 7C), RCC4 VHL^−/−^ (Figures 7D) and Cos-7 cells (Figure 7E). Significantly, silencing of CHIP prevented degradation of HIF-1α under high glucose and hypoxia (Figure 7F), emphasizing the putative relevance of CHIP-mediated degradation of HIF-1α in pathophysiological conditions such as diabetes.

## Discussion

**Figure 8:**
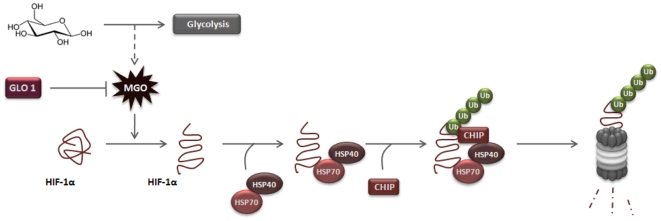
Proposed model for MGO-dependent degradation of HIF-1α. MGO induces modifications on HIF-1α protein (as for example, formation of MG-H1 adducts) and promotes increased association of Hsp40/70 to HIF-1α. This association leads to recruitment of CHIP, which promotes polyubiquitination and proteasomal degradation of HIF-1α. MGO-induced degradation of HIF-1α is activated under high glucose and is inhibited by overexpression of glyoxalase I.

In this study we elucidated a new pathway for degradation of HIF-1α. Data presented in this paper is consistent with a molecular mechanism in which HIF-1α-modification by MGO leads to increased association with the molecular chaperones Hsp40/70. This, in turn, recruits CHIP, leading to the ubiquitination and proteasome-dependent degradation of HIF-1α (Figure 8). We further showed that this mechanism is independent on the recruitment of pVHL and does not require hydroxylation of HIF-1α. This new mechanism is triggered by accumulation of MGO and is likely to have a significant physiological impact in conditions with increased availability of glucose, such as diabetes. Indeed, we showed that exposure of cells to high glucose leads to a sustained increase in the intracellular levels of MGO, which were sufficient to activate the degradation of HIF-1α. Moreover, scavenging of intracellular MGO by overexpressing glyoxalase I prevented degradation of HIF-1α under high glucose. Additionally, MGO-induced degradation of HIF-1α led to decreased transcriptional activity of HIF-1 and decreased expression of VEGF under hypoxia. Significantly, we also observed that data obtained in cell culture systems is consistent with observations in retinas of diabetic animals. Indeed, increased availability of MGO in the retina of Goto-Kakizaki rats was accompanied by decreased levels of HIF-1α and VEGF, as well as increased levels of apoptotic markers and enhanced vascular permeability [34]. The reduction of VEGF expression under high glucose and in response to hypoxia was also described before [22], [35]. The authors suggested that increased levels of MGO under hyperglycemia induce HIF-1α [22] and p300 modifications [35], which were sufficient to disrupt the interaction between HIF-1α/HIF-1β and HIF-1α/p300, leading to loss of HIF-1 transcriptional activity and poor response to hypoxia. Although this and other modifications induced by MGO cannot be excluded and are likely to account for the plethora of noxious effects of MGO in cells, our study provides robust data that elucidates an independent molecular mechanism whereby MGO can lead to proteasomal degradation of HIF-1α mediated by CHIP. It is perhaps interesting to note that the two observations are not inconsistent. Indeed, it is plausible that the decreased interaction between HIF-1α/p300 and HIF-1α/HIF-1β in the presence of MGO increases the amount of modified and monomeric HIF-1α that is available to undergo degradation through a CHIP-mediated pathway.

In the canonical mechanism for activation of HIF-1, HIF-1α is believed to be inherently stable under hypoxia and pVHL is thought to be virtually the single ubiquitin ligase targeting HIF-1α for proteasomal degradation under normoxia. Over the last five years, a number of reports suggested that HIF-1α might be destabilized under hypoxia and that interaction of HIF-1α with ancillary proteins might promote its degradation. In many instances the mechanism and molecular events that underlie HIF-1α targeting for pVHL/oxygen-independent degradation are not well understood. Nevertheless, a few proteins were shown to interact with HIF-1α promoting its proteasome-dependent degradation and some proteins were identified as putative ligases or components of ubiquitin ligase complexes that are able to ubiquitinate HIF-1α, promoting its proteasomal degradation. These include SMURF2, p53/Mdm2 complex, RACK1 and HAF [36], [37], [38], [39], [40]. Of significance for the results discussed here is a report that p53 is not involved in destabilization of HIF-1α in high glucose, since destabilization of HIF-1α is still observed in cells deficient in p53 when exposed to high glucose [7]. More recently, it was also shown that HIF-1α can be destabilized by a mechanism that is independent on both pVHL and p53 [41]. This pathway is activated in response to histone deacetylase inhibitors (HDACIs) and appears to involve destabilization of the Hsp70/90 chaperone axis resulting in proteasomal degradation of HIF-1α [41].

To the best of our knowledge, there are very few studies unequivocally elucidating ubiquitin ligases that target HIF-1α for degradation in a VHL-independent manner. In this paper, we do elucidate an entirely novel mechanism for ubiquitination and degradation of HIF-1α in a VHL-independent manner. Significantly, this alternative pathway is activated in response to intracellular accumulation of MGO and involves recruitment of Hsp40/70 and ubiquitination of the transcription factor by the E3 ligase CHIP. However, we do not exclude the possibility that this pathway might be activated by other forms of stress nor do we exclude a function for CHIP-dependent degradation of HIF-1α under physiological or pathophysiological conditions. Indeed, while the present work was in progress, it was reported that Hsp70 and CHIP can mediate ubiquitination and degradation of HIF-1α under prolonged hypoxia, consistent with the observation that prolonged hypoxia led to a decrease in HIF-1α protein levels [42].

CHIP has a critical role in protein quality control by ubiquitinating misfolded or otherwise damaged proteins through interaction with molecular chaperones [43], [44]. The observation that CHIP is also able to ubiquitinate HIF-1α reveals a new function for CHIP and an unanticipated association between high glucose, the ubiquitin-proteasome pathway and cell response to hypoxia.

It is conceivable that the mechanism reported in this manuscript is involved in MGO-induced downregulation of other cellular proteins. However, based on the present data and on the current state of art it seems prudent neither to over-generalize the mechanism nor to restrict it to HIF-1α. For example, MGO was shown to downregulate the phosphorylation of STAT3 and decrease its transcription activity [45]. Moreover, MGO was also shown to modify the co-repressor mSin3A, which results in increased recruitment of O-GlcNAc-transferase, with consequent increased modification of the transcription factor Sp3 by O-linked N-acetylglucosamine [46]. MGO is also known to suppress TNF-α-induced NF-κB activation by inhibiting NF-κB/DNA binding [47]. To the best of our knowledge, the effect of MGO on these and other transcription factors did not result in increased protein degradation. Consistently, we showed in this study that the transcription factor Nrf-2 is not downregulated by MGO. However, it is still possible that some transcription factors or other proteins may be downregulated in response to MGO. Indeed, there are a few examples where treatment with MGO was shown to lead to proteasome-dependent degradation of specific substrates. For example, it was shown that Raf-1 protein-serine/threonine kinase is degraded by an ubiquitin-proteasome-dependent mechanism in response to MGO [15]. Moreover, CML-modified proteins in glyoxal-treated cells were shown to be substrates for ubiquitin conjugation, suggesting a potential mechanism by which these modified proteins may be marked for degradation [48]. These observations raise the possibility that the ubiquitin-proteasome pathway might be implicated in degradation of proteins modified by MGO and presumably by other glycation products, which typically accumulate in diabetes.

The results reported in this paper are consistent with a number of sparse results and provide a rationale for observations such as that hyperglycemia inhibits hypoxia-induced stabilization of HIF-1α. Moreover, we have identified CHIP as the ubiquitin ligase that promotes proteasomal degradation of HIF-1α in the presence of MGO. Significantly, loss of function studies, using shRNAs against CHIP, clearly showed that endogenous CHIP is required to target HIF-1α for proteasomal degradation in the presence of physiologically relevant levels of MGO. This observation highlights the importance of this pathway under physiological conditions, as well as in pathologies associated with high glucose and/or disruption of cell response to hypoxia, including diabetes.

Although we did not address directly the modifications induced by MGO on HIF-1α that recruit Hsp40/70, we suggest that MGO-induced modification of HIF-1α (for example, by forming MG-H1 adducts) possibly leads to unfolding and exposure of hydrophobic residues at the surface of the protein. These hydrophobic patches are, most likely, recognized by the molecular chaperones Hsp40/70, which subsequently recruit CHIP to induce ubiquitination of HIF-1α and its subsequent degradation. The identification of the modified residues, as well as the type of MGO-induced modifications (i.e. by mass spectrometry), are interesting issues to pursue in the future and will certainly provide additional clues on the mechanism underlying the binding of Hsp40/70 to HIF-1α.

## Materials and Methods

### Cells Culture and Treatments

The ARPE-19 cells (LGC Promochem, Teddington, UK) were cultured in Dulbecco's modified Eagle's medium/Ham's F12 (DMEM:F12; 1∶1) supplemented with 10% fetal bovine serum (FBS), antibiotics (100 U/ml penicillin, 100 µg/ml streptomycin and 250 ng/ml amphotericin B) and GlutaMax (1x). The renal carcinoma cell line RCC4 VHL^-/-^ was grown in RPMI 1640 medium supplemented with 10% FBS, antibiotics and glutamine and was kindly provided by Dr. C. Buys (University Medical Center of Groningen, Netherlands). The Cos-7 cells (LGC Promochem, Teddington, UK) were cultured in DMEM supplemented with 10% FBS, antibiotics and glutamine. All media, GlutaMax and non-essential amino acids were purchased from Invitrogen (Carlsbad, CA, USA). When appropriate, cells were treated with the following agents: 250 µM cobalt chloride (CoCl_2_, Sigma-Aldrich), 100 µM - 3 mM methylglyoxal (MGO, Sigma-Aldrich, St. Louis, MO, USA), 10 µM epoxomicin (Boston Biochem, Cambridge, MA, USA), 20 µM MG132 or Z-LLL-CHO (Calbiochem, San Diego, CA, USA) and 10 µM MG262 or Z-Leu-Leu-Leu-B(OH)_2_ (Boston Biochem, Cambridge, MA, USA). Hypoxic treatments (5%CO_2_, 2%O_2_, 93% N_2_, 37°C) were done in a Nuaire N4950E incubator with gas control (Nuaire, Plymouth, MN, USA).

### Western Blot Analysis

After treatment, cells were washed twice in phosphate-buffered saline (PBS) solution, denatured with 2x Laemmli buffer, boiled at 100°C and sonicated. Whole cell extracts were resolved by SDS-PAGE and electrophoretically transferred onto polyvinylidene fluoride (PVDF) membranes (GE Healthcare Bio-Sciences, Uppsala, Sweden). The membranes were blocked with 5% nonfat milk in TBS-T and probed for various proteins. Immunoreactive bands were visualized with ECL (GE Healthcare Bio-Sciences, Uppsala, Sweden). The following antibodies were used: mouse anti-HIF-1α clone H1alpha67 1∶500 (Abcam, Cambridge, UK), mouse anti-actin clone C4 1∶1,000 (Millipore-Chemicon, Billerica, MA, USA), mouse anti-V5 tag clone 2F11F7 1∶2,000 (Invitrogen, Carlsbad, CA, USA), mouse anti-ubiquitin clone P4D1 1∶1,000 (Covance, Princeton, NJ, USA), mouse anti-ubiquitin clone FK1 1∶1000 (Biomol-Enzo Life Sciences, Farmingdale, NY, USA), mouse anti-c-myc clone 9E10 1∶500 (Zymed-Invitrogen, Carlsbad, CA, USA), mouse anti-Hsp70 clone C92F3A-5 1∶300 (Stressgen-Enzo Life Sciences, Farmingdale, NY, USA), mouse anti-CML clone CMS-10 1∶500 (TransGenic INC., Kumamoto, Japan), mouse anti-MG-H1 1∶500 (originally provided by Dr. M. Brownlee from the Albert Einstein College of Medicine, New York, NY, USA) [46], rabbit anti-HA 1∶200 (Zymed-Invitrogen, Carlsbad, CA, USA), rabbit anti-Hsp40 1∶2,000 (Stressgen-Enzo Life Sciences, Farmingdale, NY, USA), goat anti-STUB1 1∶500 (Abcam, Cambridge, UK) and horseradish peroxidase-conjugated secondary goat anti-mouse, goat anti-rabbit and rabbit anti-goat 1∶7,500 (Bio-Rad, Hercules, CA, USA).

### Determination of Intracellular Concentration of Methylglyoxal by HPLC

After the treatments, ARPE-19 cells (7×10^5^) cultured in 60×15 mm plates were washed twice with ice-cold PBS, scraped in 100 µl 0.1 M acetic acid and homogenized by sonication. The intracellular concentration of MGO was determined according to previously reported method [49]. Briefly, 50 µl of sample was mixed with 200 µl of 0.1 M potassium phosphate buffer pH 7.0, 200 µl of ethanol and 50 µl of freshly prepared derivatization 20 mM 1,2-diamino-4,5-dimethoxybenzene reagent (DDB; Invitrogen, Carlsbad, CA, USA) dissolved in 10 mM HCl. The mixture was incubated at least four hours at room temperature and then centrifuged for 10 minutes at 16,000 g. Subsequently, MGO was determined by reverse-phase HPLC. The HPLC system consisted of a L-6200A Intelligent Pump, a F-1080 Fluorescence Detector (Hitachi, Tokyo, Japan) and a Waters μBondapak™ C18 column (10 µm, 3.9×300 mm) (Waters, Milford, MA, USA). Mobile phase A was a mixture of 10 mM potassium phosphate buffer (pH 3.0) and acetonitrile (90/10, v/v). Mobile phase B consisted of a mixture of acetonitrile and water (70/30, v/v). Samples (20 µl) were injected and separation was performed with a linear gradient from 0-100% mobile phase B over 28 minutes. The flow rate was set at 1.0 ml/min and fluorescence detection was performed with excitation and emission wavelengths of 352 nm and 385 nm, respectively. MGO was determined by integration of peak areas using appropriate external standards.

### Metabolic labeling

ARPE-19 cells cultured in 60×15 mm plates were incubated in methionine- and cysteine-free DMEM for 40 minutes and subsequently washed twice with ice-cold PBS. L-[^35^S] methionine/cysteine (PerkinElmer, Waltham, MA, USA) was then added to a final concentration of 100 µCi/ml and cells were incubated for 4 hours under hypoxia. After metabolic labeling, the radioactive medium was removed and cells were washed twice with PBS. Cells were then recultured in complete medium containing ten fold excess of methionine/cysteine and treated as mentioned in legend of each figure. Cells were harvested at different time points in 100 µl of 0.5% NP-40 lysis buffer [50 mM Tris-HCL pH 7.4, 1501 mM NaCl, 10 mM IOD, 2 mM PMSF, 20 mM Na_3_MoO_4_ and protease inhibitor cocktail (Roche Applied Science, Indianapolis, IN, USA)]. Samples were pre-cleared with 25 µl of Protein G Sepharose (GE Healthcare Bio-Sciences, Uppsala, Sweden) for 30 minutes and then HIF-1α was immunoprecipitated as described below. Proteins were resolved by SDS-PAGE, the gels were dried and radiolabeled HIF-α protein was assessed by autoradiography.

### Immunoprecipitation

Cells cultured in 60×15 mm plates were washed twice with PBS, scraped off the dishes and collected in ice-cold PBS. Pellets were resuspended in 100 µl of lysis buffer (50 mM Tris-HCl pH 7.4, 150 mM NaCl, 10 mM IOD, 2 mM PMSF, 20 mM Na_3_MoO_4_, 0.5% NP-40 and protease inhibitor cocktail) and incubated for 30 minutes on ice. Following centrifugation at 16,000 g for 10 minutes supernatants were transferred to new tubes and 2.5 µg of anti-HIF-1α or anti-c-myc were added. The reaction was incubated overnight at 4°C with gentle agitation. Thereafter, 50 µl of protein G–Sepharose (GE Healthcare Bio-Sciences, Uppsala, Sweden) were added and incubations proceeded at 4°C for 2 hours. Beads were washed 3 times with lysis buffer and the immunoprecipitated proteins were denatured with 2x Laemmli buffer and boiled at 100°C. Samples were loaded on SDS-PAGE and western blot analyses were performed. IP controls were carried out both with no antibody and with an irrelevant mouse IgG1 antibody (for example anti-GFP).

### Immunocytochemistry

Cells were grown on coverslips and subjected to the relevant treatments. Cells were subsequently washed twice in PBS and fixed with 4% paraformaldehyde (PFA) for 10 minutes. The fixed cells were permeabilized with 1% Triton X-100 (v/v) for 10 minutes and blocked with goat serum (1∶10) for 20 minutes prior to incubation with primary antibodies for 1 hour at room temperature. The cells were then rinsed three times with 0.02% BSA in PBS and incubated with DAPI 1∶5,000 and FITC/Texas Red-conjugated goat anti-mouse or anti-rabbit 1∶100 (Invitrogen, Carlsbad, CA, USA) for 1 hour at room temperature. The coverslips were washed with 0.02% BSA and mounted with Glycergel (Dako, Glostrup, Denmark). The cells were imaged by confocal microscopy using the MRC600 image system (Bio-Rad, Hercules, CA, USA).

### Measurement of 20S proteasome activity

Cells were washed twice with PBS, lysed with a Tris buffer (50 mM Tris pH 7.4, 1 mM DTT) and sonicated. After centrifugation (16,000 g for 10 minutes at 4°C), protein concentration was determined using the Coomassie method and 40 µg of protein was incubated with the following fluorogenic substrates: 100 µM Suc-LLVY-MCA for the chymotrypsin-like activity (Biomol-Enzo Life Sciences, Farmingdale, NY, USA); 25 µM Boc-LRR-MCA for the trypsin-like activity (Biomol-Enzo Life Sciences, Farmingdale, NY, USA); 150 µM Z-LLE-MCA for the caspase-like activity (Calbiochem, San Diego, CA, USA). The proteasome activities were monitored during 1 hour at 37°C, for periods of 5 minutes (excitation wavelength at 380 nm; emission wavelength at 460 nm). Absorbance was measured on a Biotek Synergy HT spectrophotometer (Biotek, Winooski, VT, USA), using the Gen 5 software to monitor the results (Biotek, Winooski, VT, USA).

### Plasmids

Human CHIP (GeneBank accession number NM_005861) was amplified by PCR from a human leukocyte cDNA library, while human glyoxalase I (GeneBank accession number NM_006708) was PCR-amplified from the pCMS-EGFP hGLOI vector [21]. Both amplifications were performed using flanking primers containing *Eco*RI and *Sal*I restriction sites and the sequences were cloned into pENTRV5-C2 [50], [51] to generate pENTR/V5-hCHIP and pENTR/V5-hGLOI. In order to produce hCHIP and hGLOI adenovirus, these vectors were recombined with the pAd/BLOCKiT-DEST adenoviral vector from Invitrogen using Gateway technology according to manufacturers' instructions. For this work we also used the following plasmids: pcDNA3.1 c-myc-CHIP wt, pcDNA3.1 c-myc-CHIP K30A, pcDNA3.1 c-myc-CHIP H260Q [52]; pcDNA6 Hdj1/Hsp40 wt-V5 [53]; pcDNA3 Hsp70 wt-HA [54]; pcDNA3 HIF-1α wt-V5 and pcDNA3 HIF-1α (P402A and P564A)-V5 [55]; pcDNA3 HA-hHIF1α wt [2].

### Adenoviral CHIP shRNA production and infection

For shRNA targeting of human CHIP, the oligonucleotides were annealed and ligated into pENTR/U6 according to manufacturers' instructions. The following oligonucleotides were used: shRNA1 forward 5′-CACCGGAGATGGAGAGCTATGATGACGAATCATCATAGCTCTCCATCTCC-3′; shRNA1 reverse 5′- AAAAGGAGATGGAGAGCTATGATGATTCGTCATCATAGCTCTCCATCTCC-3′; shRNA2 forward 5′-CACCGGCTATGAAGGAGGTTATTGACGAATCAATAACCTCCTTCATAGCC-3′; shRNA2 reverse 5′-AAAAGGCTATGAAGGAGGTTATTGATTCGTCAATAACCTCCTTCATAGCC-3′ (shRNA target sequence is underlined). All plasmids were verified by DNA sequencing. Site-specific recombination between pENTR286 attL sites and pAd/BLOCKiT-DEST attR sites was performed using LR clonase II (Invitrogen, Carlsbad, CA, USA), according to the manufacturer's instructions. Pac1-digested recombinant packaging vector was then transfected into a producer cell line HEK293A and recombinant viral particles were harvested after lysis of infected cells 8-10 days later [50]. The word “Mock” in the gene silencing figures refers to a control with a scrambled shRNA sequence.

### Transient Transfection

One day before transfection, cells were seeded in 60×15 mm plates in 2.5 ml of medium so that the cells were 90% confluent at the time of transfection. 4 µg of plasmid DNA were used per dish and transfections were carried out using Lipofectamine 2000 (Invitrogen, Carlsbad, CA, USA), according to the manufacturer's specifications. After transfection, cells were incubated at 37°C in a CO_2_ incubator for 24-30 hours prior to test for transgene expression. The word “Mock” in the figures refers to a control with an empty vector.

### Reporter-gene Assay

ARPE-19 cells were plated in 60×15 mm dishes at subconfluent density and transfected with pT81 HRE-luciferase [7], using Lipofectamine 2000 (Invitrogen, Carlsbad, CA, USA). The plasmid pT81/HRE-luciferase contains three tandem copies of the erythropoietin hypoxia response element (HRE) in front of the herpes simplex thymidine kinase promoter. Twenty-four hours after transfection, cells were treated as mentioned and then assayed for luciferase activity, which was measured using a LMax II 384 ROM v1.04 reader and SoftMax Pro 5 software (Molecular Devices, Sunnyvale, CA, USA), as described by the manufacturer's specifications. The different treatments to which cells were subjected had no effect on the readout of the luciferase reporter assay (assessed by transfection of pCMV luciferase).

### Determination of VEGF in supernatants

The concentration of diffusible VEGF-A_121_, _165_ in the cell culture supernatants was measured by a Quantikine VEGF enzyme-linked immunosorbent assay kit (R&D Systems, Minneapolis, MN, USA), using monoclonal antibodies directed against human VEGF. Absorbance was measured at 450 nm, with wavelength correction at 570 nm, on a Biotek Synergy HT spectrophotometer (Biotek, Winooski, VT, USA), using the Gen 5 software to monitor the results (Biotek, Winooski, VT, USA).

### Quantitative Real-Time PCR

Following the relevant treatments, total RNA was purified according to the manufacturer's specifications of Qiagen RNeasy mini kit (Qiagen, Valencia, CA, USA) and treated with RNase-free DNase I (GE Healthcare Bio-Sciences, Uppsala, Sweden). SuperScript II Reverse Transcriptase (Invitrogen, Carlsbad, CA, USA) and random hexadeoxynucleotide primers were used to synthesize cDNA. For human VEGF mRNA quantification, it was used the SYBR Green PCR master mix (Bio-Rad, Hercules, CA, USA), according to the manufacturer's instructions, and the cDNA amplification was performed using the following set of primers: hVEGF forward 5′-CAGAATCATCACGAAGTG-3′; hVEGF reverse 5′-TCTGCATGGTGATGTTGGAC-3′; 18S rRNA forward 5′-GTCTGCCCTATCAACTTTC-3′; 18S rRNA reverse 5′-TTCCTTGGATGTGGTAGC-3′. The real time PCR analyses were conducted on a ABI Prism 7000 quantitative PCR system (Applied Biosystems, Foster City, CA, USA).

### Statistical Analysis

Data are reported as the means ± standard deviation of at least three independent experiments. Comparisons between multiple groups were performed by one-way analysis of variance test (ANOVA) with Dunnet's or Tukey's multiple comparison tests, using GraphPad Prism 5.0 software (GraphPad Software, La Jolla, CA, USA). For comparison between two groups, the paired *t* test was used. In all cases, *p<*0.05 was considered significant.
